# The Metabolomic Footprint of Liver Fibrosis

**DOI:** 10.3390/cells13161333

**Published:** 2024-08-11

**Authors:** Diren Beyoğlu, Yury V. Popov, Jeffrey R. Idle

**Affiliations:** 1 Department of Pharmaceutical and Administrative Sciences, College of Pharmacy and Health Sciences, Western New England University, Springfield, MA 01119, USA; diren.beyoglu@wne.edu; 2 Division of Gastroenterology, Hepatology and Nutrition, Beth Israel Deaconess Medical Center, Harvard Medical School, Boston, MA 02215, USA; ypopov@bidmc.harvard.edu

**Keywords:** liver fibrosis, NMR, GC–MS, LC–MS, TCM, chemical constituents, molecular docking

## Abstract

Both experimental and clinical liver fibrosis leave a metabolic footprint that can be uncovered and defined using metabolomic approaches. Metabolomics combines pattern recognition algorithms with analytical chemistry, in particular, ^1^H and ^13^C nuclear magnetic resonance spectroscopy (NMR), gas chromatography–mass spectrometry (GC–MS) and various liquid chromatography–mass spectrometry (LC–MS) platforms. The analysis of liver fibrosis by each of these methodologies is reviewed separately. Surprisingly, there was little general agreement between studies within each of these three groups and also between groups. The metabolomic footprint determined by NMR (two or more hits between studies) comprised elevated lactate, acetate, choline, 3-hydroxybutyrate, glucose, histidine, methionine, glutamine, phenylalanine, tyrosine and citrate. For GC–MS, succinate, fumarate, malate, ascorbate, glutamate, glycine, serine and, in agreement with NMR, glutamine, phenylalanine, tyrosine and citrate were delineated. For LC–MS, only β-muricholic acid, tryptophan, acylcarnitine, *p*-cresol, valine and, in agreement with NMR, phosphocholine were identified. The metabolomic footprint of liver fibrosis was upregulated as regards **glutamine**, **phenylalanine**, **tyrosine**, **citrate and phosphocholine**. Several investigators employed traditional Chinese medicine (TCM) treatments to reverse experimental liver fibrosis, and a commentary is given on the chemical constituents that may possess fibrolytic activity. It is proposed that molecular docking procedures using these TCM constituents may lead to novel therapies for liver fibrosis affecting at least one-in-twenty persons globally, for which there is currently no pharmaceutical cure. This in-depth review summarizes the relevant literature on metabolomics and its implications in addressing the clinical problem of liver fibrosis, cirrhosis and its sequelae.

## 1. Introduction

The excessive accumulation in the liver of extracellular matrix proteins, particularly collagen, is the hallmark of liver fibrosis and is a wound-healing response to liver injury. Liver fibrosis in developed nations is caused mainly by alcohol abuse, chronic HCV and HBV infection and the rising prevalence of metabolic dysfunction-associated steatotic liver disease (MASLD) and metabolic dysfunction-associated steatohepatitis (MASH) due to obesity epidemics [1,2]. MASH is of particular concern worldwide, rapidly becoming the leading cause of liver-related morbidity and mortality. MASH may be present in 10–25% of patients with hepatic steatosis due to MASLD, a condition found in most obese individuals [3]. According to the recent epidemiological data, the global prevalence of MASLD reached an alarming 25.24% [4], which corresponds to an estimated 100 million people in the US alone, and rising incidence in young individuals [5]. Multiple retrospective studies have suggested that liver fibrosis is the most critical biological determinant of human MASLD and is associated with the disease progression and development of complications [6,7,8]. Most recently, a large prospective multicenter study of 1773 patients confirmed that, of all histological disease characteristics, only advanced fibrosis is strongly associated with an increased risk of liver-related complications and death in NAFLD/NASH [9], strongly suggesting that understanding and therapeutically addressing liver fibrosis is of central importance in MASLD. Moreover, chronic liver diseases with no curative treatment, such as MASLD and HBV, are major contributors to the rising incidence of primary cancers, a clinical sequalae of liver fibrosis [10,11]. Although genomic integration and chronic inflammation play major roles in driving hepatocellular carcinoma (HCC) in HBV, liver fibrosis is a significant factor in reducing the immune response against HBV [12].

Early investigations of liver fibrosis were conducted in a wide range of laboratory animals with many predisposing procedures, such as dietary restriction [13], the effect of aging [14], infection with *Schistosoma mansoni* [15] or *Schistosoma japonicum* [16], immunization with heterologous sera and other foreign proteins [17] or with hypophysectomy [18,19] and/or thyroidectomy [19]. Other fibrogenic procedures involved the administration of hepatotoxins such as chloroform [20], carbon tetrachloride (CCl_4_) [21], ethionine [22], thioacetamide (TAA) [23], galactosamine [24] or *Salmonella typhosa* endotoxin [25]. It was apparent from these early investigations that diet played a highly significant role in the response of the animal liver to chemical toxins. Specifically, a diet high in fat fed to dogs led to greater chloroform hepatotoxicity than a diet rich in protein [20]. Similarly, “all-fat” diets preceding CCl_4_ administration to dogs resulted in maximal injury to liver parenchyma. In contrast, high-protein diets were considered protective and, moreover, high-carbohydrate diets afforded a striking defense against liver injury by CCl_4_ [26]. Studies then turned to the identification of lipotropic chemical constituents of the diet that might protect against liver injury. Purified lecithin had been shown to prevent the deposition of fat in the livers of normal rats; therefore, experiments were then undertaken to identify the specific moiety of lecithin that was responsible for this effect. This was found to be choline, the inclusion of as little as one percent of which in the diet was found to prevent the hepatic infiltration of lipids [27]. The deposition of both triglycerides and cholesterol esters was prevented by dietary choline [28,29]. It was suggested that choline in some way accelerated the oxidation of both fatty acids and cholesterol by the liver, presumably by β-oxidation [28]. Therefore, as early as the 1930s, it was presupposed that metabolism was a component in the mechanism of hepatic injury.

A diet low in protein promoted an extensive fat infiltration of the liver, and this could be prevented by the addition of the milk protein casein. The lipotropic activity of casein was considered to be due to its content of the amino acid methionine and to the presence of choline in the diet [30]. The lipotropic effects of methionine and choline could be counteracted by the presence of dietary cystine or proteins rich in cystine, such as peanut protein. Interestingly, the addition of cystine to an experimental diet exerted a strong protective effect against the development of hepatic necrosis, while appearing to enhance the appearance of fibrosis. It was further proposed that the presence of cystine and choline in the diet was required for the synthesis of methionine, which was the active lipotropic agent [31]. Therefore, the administration of choline plus cystine or of methionine was highly effective in preventing experimental injury to the liver. The exposures causing or preventing liver fibrosis are shown in Table 1.

These early descriptions of dietary and other circumstances leading to fat infiltration, necrosis, fibrosis and cirrhosis were undoubtedly useful. However, a map of the underlying biochemical changes would be necessary to understand more fully the pathobiology and to develop interventional strategies. The question arose as to whether or not the metabolism of cystine to inorganic sulfate was the cause of liver necrosis and cirrhosis. Experiments were conducted in rats that were fed diets sufficient to cause portal necrosis and, ultimately, liver cirrhosis. In addition, groups were administered cystine, methionine or cysteic acid and their urinary excretion of sulfate determined. Both cystine and methionine led to high urinary sulfate excretion, while cysteic acid did not. Both the cystine and cysteic acid administrations resulted in hepatic necrosis and cirrhosis, but that of methionine did not. This biochemical investigation demonstrated that the metabolic formation of sulfate was unrelated to hepatoxicity [32]. A further investigation of methionine found that it could inhibit ductal cell proliferation and fibrogenesis caused by ethionine [33] and also prevent ethionine-induced liver carcinoma [34] in rats. The consensus at the time was that methionine could prevent most of the known effects of ethionine and that methionine was required for choline synthesis [34]. The first report of *S*-adenosylmethionine (SAM), an “activated methionine” formed enzymically in the liver by the reaction of methionine with ATP [35,36], was to have a major biological impact. SAM is one of the most versatile molecules in living systems, involved in myriad biochemical reactions, second only to ATP [37]. SAM is the methyl donor for the preponderance of methyltransferases that modify DNA, RNA, histones and other proteins, and the vast majority of these processes occur in the liver [37]. Ethionine can be metabolized to *S*-adenosylethionine, which is capable of transethylation reactions leading to abnormal metabolic products and depleting the cell of ATP [38]. This, in turn, results in an up to 95% reduction in hepatic RNA synthesis [39,40], leading to impaired protein synthesis [40]. DNA synthesis in rat liver was also affected by ethionine with the formation of 7-ethylguanine bases [41]. In addition, the incorporation of ethionine led to abnormal proteins, particularly in the intestinal mucosa, liver and kidney of rats [42]. These effects were believed to underlie the toxic effects of ethionine on the liver. A metabolic component of liver injury was clearly significant.

The production of α-smooth muscle actin (α-SMA) and the extracellular matrix (ECM) protein collagen type I in hepatic fibrosis derives from hepatic stellate cells (HSCs) that have been activated from lipocytes to myofibroblasts by TGF-β1, lipid peroxidation or oxidative stress [43,44,45]. The production of ECM is dependent on mitochondrial energy metabolism [46]. Monitoring metabolic pathways in the liver with respect to fibrosis would appear to be a fruitful means of increasing the understanding of the mechanisms of fibrogenesis. The past two decades have witnessed an eruption in the literature of global metabolite determinations by metabolomics, in particular, using mass-spectrometry-based methodologies and approaching 25,000 reports. We have previously reported aspects of metabolomics in relation to liver disease [47,48,49,50,51,52,53,54]. 

## 2. Metabolomics

Diagnostic “urine charts” have been used since the Middle Ages, relating the taste, smell and color of urine to various diseases. Such changes were likely to be metabolic in origin [55]. The field of metabolomics has its origins in the study of the functional genomics of the yeast *Saccharomyces cerevisiae*, with the first use of the term “metabolome” in September 1998 [56,57]. The metabolome was seen as complementing the transcriptome and proteome as outputs of the yeast genome [56]. Oliver Fiehn envisaged a comprehensive analysis of the metabolome to comprehend the metabolic effects of altering a single gene in plants and named this process “metabolomics” [58]. Earlier, Nicholson and colleagues had introduced the term “metabonomics”, which they defined as “the quantitative measurement of the dynamic multiparametric metabolic response of living systems to pathophysiological stimuli or genetic modification” [59]. Today, the differences between metabolomics and metabonomics are little more than semantic, both employing pattern recognition methods to interpret resulting complex chemical–analytical data sets. Using metabolomics, it is commonplace to screen biofluids, such as urine or serum, for their metabolite composition in disease cases and matched controls in order to find biomarkers for risk prediction [60]. Furthermore, alterations in biological pathways can be detected that provide insights into disease mechanisms [61]. The various analytical methodologies employed in metabolomics, nuclear magnetic resonance spectroscopy (NMR), gas chromatography–mass spectrometry (GC–MS) and liquid chromatography–mass spectrometry (LC–MS) have been discussed [48,55,62] and the difference between targeted and untargeted metabolomics explained [62]. NMR, GC–MS and LC–MS analyses have been applied in combination for the investigation of liver disease [63,64,65].

### 2.1. Early Studies Using NMR-Based Metabonomics

Fifty years ago, the study of the liver was rooted in morphology with no biochemical analysis until the application of proton nuclear magnetic resonance spectroscopy (^1^H NMR) to the investigation of experimental liver tumors [66,67]. This novel approach was prompted by earlier observations that gastric and colonic tumors harbored lower calcium but higher potassium concentrations [68,69,70], which were interpreted as involving changes in the water content of the malignant issues [66,67]. Using ^1^H-NMR, the spin–lattice relaxation time T_1_ (time for the relaxation of excited ^1^H nuclei to the lower energy ground state), which is influenced by tissue water content, was reported to be 2.5-times greater for rat hepatomas than normal liver specimens. This was interpreted as being due to the increased motional freedom of water molecules in neoplastic tissue [67].

After the definition of metabonomics in 1999 [59], there was a flurry of studies investigating the biochemical perturbations of the liver using NMR-based metabonomics [71,72,73,74]. An early study of fibrosis was conducted in rats administered TAA for which aqueous extracts of the liver showed elevations in branched-chain amino acids (BCAAs), lactate, alanine, acetate, acetoacetate, glutamine and trimethylamine (TMA) [75]. Similarly, rats treated with aflatoxin B_1_ (AFB_1_) demonstrated elevations in glucose, amino acids and choline metabolites (choline, phosphocholine and glycerophosphocholine) in plasma but a reduction in plasma lipids. AFB_1_ also induced the elevation of liver lipids, amino acids (tyrosine, histidine, phenylalanine and the BCAA), choline and nucleic acid metabolites (inosine, adenosine and uridine) together with a reduction in hepatic glycogen and glucose [76]. 

The serum of patients with advanced fibrotic disease (liver cirrhosis (LC) and hepatocellular carcinoma (HCC)) using ^1^H NMR revealed increases in aromatic amino acids (AAA) but decreases in choline and BCAA in LC that were translated also to HCC compared to healthy subjects [77]. ^1^H high-resolution magic-angle-spinning (HR-MAS) NMR spectroscopy [78,79] has been used to investigate the metabolic profiles of liver needle biopsies from patients with chronic hepatitis and LC mostly due to hepatitis C virus (HCV) infection. A partial least-squares-discriminant analysis (PLS-DA) score plot [48] was able to distinguish the metabolic profiles of cirrhosis and non-cirrhosis liver biopsies [80]. NMR-based metabolomics was used to investigate chronic liver failure (CLF) in cirrhosis. Serum concentrations of lactate, pyruvate, glucose, amino acids and creatinine were higher in patients with severe CLF than mild CRF [81]. Compensated and decompensated cirrhoses have been similarly compared [82]. These early NMR-based metabolomic investigations were able to delineate relatively few altered metabolites in various stages of fibrotic liver disease, in particular, BCAA, AAA and choline and its derivatives, lactate, pyruvate and glucose. The NMR studies are summarized in Table 2. However, these observations have not contributed to a better understanding of the mechanisms of liver fibrosis or its progression to cirrhosis and HCC. 

### 2.2. Recent Studies Using ^13^C and ^31^P NMR-Based Metabonomics

^13^C is a stable isotope of carbon with a natural abundance of 1.1% and has a spin quantum number of ½, which makes it an NMR-active nucleus. In contrast, the most abundant carbon isotope ^12^C (natural abundance 98.9%) has a spin quantum number of 0 and, therefore, cannot be detected using NMR. This characteristic can be exploited to probe metabolic processes in vivo by infusing ^13^C-enriched substrates into patients under investigation and then following the incorporation of the ^13^C atoms into the metabolic products [83]. ^31^P is the only stable isotope of phosphorus with a natural abundance of 100%. It has a spin quantum number of ½, making it suitable for NMR spectroscopy. ^31^P NMR is much less sensitive than ^1^H NMR but more sensitive than ^13^C NMR.

The past decade has witnessed the increasing utilization of ^13^C and ^31^P NMR spectroscopy and magnetic resonance imaging (MRI) in the investigation of molecular alterations in liver disease, including various stages of fibrosis. The detection of molecular changes in fibrosis using MRI was established during the past fifteen years [84]. 

More recently, one uncommon methodology involving hyperpolarized ^13^C MRI has been developed, which can be used to image healthy and diseased tissues and would involve an injection of the patient with so-called “hyperpolarized” [1-^13^C]pyruvate [85,86,87]. The hyperpolarization of a [1-^13^C]pyruvate solution is typically accomplished after the addition of an electron paramagnetic agent (typically trityl radical) using a DNP (dynamic nuclear polarization) polarizer with a magnetic field strength of 3.35 T (Tesla) and a microwave source of 94 GHz. During the hyperpolarization procedure of 15 min to 6 h, the sample is held at 1.4 K using liquid helium, resulting in an average polarization of ≈20%, i.e., an alignment of ≈20% of the ^13^C nuclear spins [88]. For obvious reasons, this procedure is not in common clinical practice. This technology allows for a >10,000-fold increase in sensitivity over conventional ^13^C NMR [89]. Nevertheless, hyperpolarized [1-^13^C]pyruvate administration to mice with different stages of chemically induced liver fibrosis has been employed to define the footprints of fibrosis progression that might be possible to translate to clinical investigations. In particular, ratios of [1-^13^C]lactate/pyruvate, [1-^13^C]lactate/total carbon, [1-^13^C]alanine/pyruvate and [1-^13^C]alanine/total carbon were significantly higher in both the mild and severe fibrosis mouse groups than in the normal control group. [1-^13^C]alanine/pyruvate and [1-^13^C]alanine/total carbon were significantly higher in the severe fibrosis group than in the mild fibrosis group [88]. These findings suggest that the transamination of pyruvate to alanine by alanine aminotransferase (ALT) and pyruvate to lactate by lactate dehydrogenase (LDH) is elevated throughout fibrosis progression, which is generally consistent with elevated serum ALT levels in a large retrospective observational study of 771 liver biopsies [90] and the correlation of LDH levels with the presence of fibrosis in HCC patients [91]. It would appear unlikely that the introduction of hyperpolarized ^13^C MRI could provide any clinical benefit beyond the routine determination of serum ALT and LDH levels.

MRI machines used in hospitals today generally contain a 0.5 to 3 T magnet. Improved resolution for ^31^P MRI images can be obtained using a 7 T magnet that generates a five-times stronger magnetic field. To put this in context, a fridge magnet is 0.01 T and a junkyard magnet is 1 T [92]. Organophosphorus molecules are useful biomarkers for liver disease with phosphatidylcholine, nicotinamide adenine dinucleotide (NAD^+^) and uridine diphosphoglucose (UDPG) measurable by ^31^P 7 T MRS (magnetic resonance spectroscopy) in 10 healthy volunteers and 11 patients with cirrhosis. Inorganic phosphate and phosphatidylcholine concentrations were significantly lower in patients, while glycerophosphatidylethanolamine concentrations were significantly higher [93]. Elevated phosphatidylethanolamine (PE) is probably due to the reduced expression of PE *N*-methyltransferase 2 (PEMT2) enzyme, which catalyzes the conversion of PE to phosphatidylcholine in the liver when dietary choline supply is low [94,95], as substantiated using *Pemt2* null mice [96]. 

### 2.3. Recent Studies Using ^1^H NMR-Based Metabolomics

Non-alcoholic fatty liver disease (NAFLD), now generally termed metabolic dysfunction-associated steatotic liver disease (MASLD) [97,98], is the most common liver disease worldwide, with a prevalence of 30% and growing, and is the leading cause of liver-related morbidity and mortality [99,100]. There is a high prevalence of advanced liver fibrosis in patients with MASLD [101]. The main driver of fibrosis in MASLD is inflammation, but this is often difficult to evaluate because of the dynamic nature of the disease and its poor correlation with standard biomarkers [102]. Liver biopsy can play a role but is invasive, costly, time-consuming and potentially painful, and it suffers from interobserver diagnostic variability [102,103]. Consequently, noninvasive methods have emerged for the evaluation of the presence and extent of liver fibrosis, in particular, transient elastography (TE) and magnetic resonance elastography (MRE), which determine liver stiffness [104,105]. Mac-2-Binding Protein Glycosylation Isomer (M2BPGi) has also been compared favorably with TE in the evaluation of liver stiffness [106]. It is hardly surprising that other noninvasive biomarkers have emerged as candidates to replace liver biopsy in the determination of the degree of liver fibrosis, in particular, using metabolomics.

The METAVIR scale scores fibrosis on four levels—F0, the absence of fibrosis or scaring; F1, portal tract fibrosis without septa formation (mild to moderate fibrosis); F2, portal tract fibrosis with infrequent/rare septa formation (significant fibrosis); F3, numerous septa but no cirrhosis (severe/advanced fibrosis); F4, complete nodules with advanced scaring: cirrhosis [107,108]. The question is as follows: What metabolomic methodologies, if any, can determine the severity of fibrosis? An investigation of HCV-infected patients with F0 and F4 fibrosis was conducted using 600 MHz (14.1 T magnet) ^1^H NMR spectroscopy on their sera. Choline, acetoacetate and low-density lipoprotein were all significantly diminished in cirrhosis compared to F0 fibrosis [109]. Another 600 MHz ^1^H NMR study of HCV patients with fibrosis (F3–F4) reported a complex serum metabolic profile comprising 21 metabolites, which included eleven amino acids, five carboxylic acids, four purines and urea [110]. None of these corresponded to the aforementioned study [109]. A further study conducted on the sera of chronic hepatitis C (CHC) patients with fibrosis and healthy controls using 800 MHz ^1^H NMR (18.8 T magnet) and supervised PLS-DA multivariate data analysis was reported. Here, CHC patients displayed reduced serum proline, serine, valine, glutamine and creatinine with elevated fucose, carnitine, lysine, 3-hydroxybutyrate, ornithine, glycerol, methionine, methanol and isopropanol relative to the healthy controls. Described by the authors as the “metabolic fingerprint of chronic hepatitis C progression,” these findings were further interpreted by pathway enrichment analysis using MetaboAnalyst and the KEGG ––database. This resulted in six metabolic pathways being affected in CHC, including glycine, serine and threonine metabolism, glycerolipid metabolism, arginine, proline metabolism, aminoacyl-tRNA biosynthesis, cysteine and methionine metabolism [111]. Interestingly, the results using 800 MHz spectroscopy did not correspond to the findings of either of the 600 MHz spectroscopy studies [109,110,111]. The lack of congruence of these three studies underlines the importance of the clinical description of patients rather than the NMR technology employed. It must be stated that none of these three investigations addressed the important issue of fibrosis progression biomarkers in relation to the METAVIR F0 to F4 classification of fibrotic disease.

A Danish study using 600 MHz ^1^H NMR investigated 90 patients with chronic hepatitis B (CHB) infection over a 10-year period. These authors classified their patients as Phase I (immune tolerance), Phase II (immune clearance), Phase III (inactive carrier) and Phase IV (reactivation) using HBeAg and HBeAb levels and, only to a minor extent, liver stiffness measured by fibroelastography using FibroScan [112]. When the Echosens company in Paris, France, introduced the FibroScan instrument in 2003 as a new noninvasive method for the assessment of hepatic fibrosis, it was claimed that the liver elasticity measurements were highly correlated (*p* < 0.0001) with the METAVIR fibrosis grade [113]. Over the years, there has been considerable debate regarding the interpretation of FibroScan data (see [114] for an editorial and [115] for a discussion of limitations, pitfalls and confounders). Moreover, a study conducted in 23 French university hospital hepatology departments concluded that FibroScan has been reported to have a high diagnostic accuracy for cirrhosis but not for significant fibrosis (METAVIR ≥ 2) in patients with chronic hepatitis B or C. The authors concluded that liver biopsy was warranted for the diagnosis of intermediate stages of fibrosis [116]. It must be noted that simple, transient elastography (TE) procedures have been enhanced through combination with MRI, producing magnetic resonance elastography (MRE) [117,118,119]. In a prospective, cross-sectional study of more than 100 patients (47, 24, 11, 13 and 8 patients with stages 0, 1, 2, 3 and 4 fibrosis, respectively), MRE was reported to be more accurate than TE in detecting liver fibrosis (stage > 1), but neither technique fared well in the discrimination of stages 1, 2, 3 and 4 [120]. 

Can metabolomic biomarkers improve upon the diagnosis of the liver fibrosis stage over these various elastography methodologies? A Canadian study of 20 CHC patients and 14 non-CHC controls found that FibroScan TE could not distinguish between controls and patients with early-stage liver fibrosis (F0–F1). However, late-stage fibrosis (F2–F4) was distinguished by TE. Furthermore, when sera were analyzed both by ^1^H NMR (700 MHz; 16.4 T magnet) and multisegment injection–capillary electrophoresis–mass spectrometry (MSI–CE–MS), the ratio of serum choline to uric acid provided the optimal differentiation of liver disease severity (AUC = 0.848, *p* = 0.00766) using a receiver operating characteristic (ROC) curve, which was positively correlated with liver stiffness measurements by FibroScan TE (r = 0.606, *p* = 0.0047) [121]. Beyond NMR, mass-spectrometry-based methodologies now dominate the metabolomics space. Can these procedures better define the stages of liver fibrosis progression? The following text regarding metabolomic investigations in liver fibrosis will attempt to answer this question.

**Table 2:** Biochemical changes in liver fibrosis discovered by NMR-based metabolomics, MRI and MRS.

Species	Fibrogen	Tissue	Upregulated Metabolites	Downregulated Metabolites	Ref.
rat	thioacetamide	liver	BCAA, lactate, alanine, acetate, acetoacetate, glutamine, TMA	-	[75]
rat	thioacetamide	serum urine	phenylalanine, *N*,*N*-dimethyl glycine, *O*-acetyl glycoprotein, *N*-acetyl glycoprotein choline 2-hydroxybutyrate, 3-hydroxybutyrate, adipate	-	[122]
rat	aflatoxin B_1_	plasma liver	glucose, amino acids, choline, phosphocholine, glycerophosphocholine lipids, tyrosine, histidine, phenylalanine, BCAA, choline, inosine, adenosine, uridine	lipids glycogen, glucose	[76]
human human human human mouse (MRS) human (MRI) human human human	HBV HCV alcohol HBV thioacetamide HCV, MASLD, ASH, AIH HCV HCV HCV	serum liver serum serum liver liver serum serum serum	acetate, *N*-acetylglycoproteins, pyruvate, glutamine, 2-oxo-glutarate, taurine, glycerol, tyrosine, 1-methylhistidine, phenylalanine glutamate, phosphocholine, phosphoethanolamine lactate, pyruvate, glucose, BCAA, methionine, glutamine, citrate, creatinine glucose, lactate [1-^13^C]lactate/pyruvate, [1-^13^C]lactate/total carbon, [1-^13^C]alanine/pyruvate, [1-^13^C]alanine/total carbon glycerophosphatidylethanolamine VLDL1, citrate, lipid, glucose/sugars, phenylalanine histidine, methionine, tyrosine, methylsuccinate, formate, propionate, 2-hydroxy-isovalerate, 2-oxoisocaproate, methylguanidine, 1,7-dimethylxanthine, caffeine fucose, carnitine, lysine, 3-hydroxybutyrate, ornithine, glycerol, methionine, methanol, isopropanol	LDL, VLDL, BCAA, acetoacetate, choline, unsaturated lipid glucose - Lipids, choline inorganic phosphate, phosphatidylcholine LDL, acetoacetate, choline, BCAA, creatinine, creatine, glutamate, glutamine, HDL, asparagine, VLDL2, lysine, arginine, glycerol, 3-hydroxybutyrate, histidine *N*-acetylglycine, asparagine, creatinine, glutamine, glycine, methylhistidine, *N*-acetylaspartate, threonine, urea, adenosine proline, serine, valine, glutamine, creatinine	[77] [80] [82] [88] [93] [109] [110] [111]

Abbreviations: MRS, magnetic resonance spectroscopy; MRI, magnetic resonance imaging; TMA, trimethylamine; LDL, low-density lipoprotein; VLDL, very-low-density lipoprotein; MASLD, metabolic dysfunction-associated steatotic liver disease; ASH, alcoholic steatohepatitis; AIH, autoimmune hepatitis.

### 2.4. Summary of NMR Studies on Liver Fibrosis

Table 2 shows the findings from eight human, three rat and one mouse investigation on liver fibrosis that employed ^1^H NMR, ^13^C NMR and ^31^P NMR, together with MRI and MRS clinical investigations. A perusal of this table reveals the highly heterogeneous findings of these studies. Even in the four human investigations where HCV was the only provoking fibrogen, the findings are quite disparate. In the three human HCV investigations that analyzed serum, one study compared HCV patients with (F4) and without (F0) fibrosis using a 600 MHz NMR spectrometer [109]. A second study also employed 600 MHz NMR to analyze serum from HCV patients with either F0-1 or F3-4 fibrosis [110]. In essence, these two investigations were very similar. However, citrate, “lipid,” “glucose/sugars” and phenylalanine were the only elevated serum constituents in the former study, while histidine, methionine, tyrosine, methylsuccinate, formate, propionate, 2-hydroxy-isovalerate, 2-oxoisocaproate, methylguanidine, 1,7-dimethylxanthine and caffeine were raised in the latter study. Furthermore, these two reports shared no elevated serum molecules in common, but both had depressed serum concentrations of creatinine, asparagine and glutamine in their fibrosis groups [109,110] (Table 2). The third study used a 800 MHz NMR spectrometer to compare sera from HCV patients with an undeclared severity of fibrosis (but without cirrhosis) with healthy controls [111]. The pattern of elevated metabolites was distinct from the other two HCV studies with the exception that methionine was elevated as in the latter study [110]. Among these elevated metabolites were reported methanol and isopropanol [111]. This is surprising because the human serum concentration of methanol is typically around 3 mmol/L [123], and isopropanol is rarely found in human blood and then at around 0.5 mmol/L [124]. In addition, the limit of detection for small molecules using ^1^H NMR is generally ~10 mmol/L [125]. This raises a query about the assignment of metabolite identities in certain ^1^H NMR studies. The unifying observation for all three HCV patient studies was simply a diminished serum concentration of glutamine. This is surprising because each of these three HCV patient investigations identified approximately 15–20 elevated or diminished serum metabolites (see Table 2).

### 2.5. Gas Chromatography–Mass Spectrometry-Based Metabolomics

The principles of gas chromatography–mass spectrometry (GC–MS) have been described in an outline by the American Chemical Society [126] and in detail in a classical textbook [127]. GC–MS combined with multivariate statistics was first popularized in plant metabolomics by Oliver Fiehn, in particular, for leaf extracts of the small cress plant *Arabidopsis thaliana* [128,129]. Based upon the ability to discriminate plant genotypes using GC–MS metabolomics [130], Fiehn characterized metabolomics as “the link between genotypes and phenotypes” [58]. Regarding liver fibrosis, many Chinese research groups have employed GC–MS metabolomics to define the metabolic changes in rats or mice administered fibrogenic chemicals such as CCl_4_ or dimethylnitrosamine (DMN) and the metabolic effects of traditional Chinese medicines (TCM) in preventing or ameliorating liver fibrosis [131,132,133,134,135,136,137,138,139,140]. In these investigations, the administration of CCl_4_ to rats resulted in a diminished urinary excretion of the amino acids leucine, glycine, proline, tryptophan and lysine and the gut microbiota metabolites benzoate, phenol and indole. Moreover, increased urinary excretion was observed for succinic acid, indole-3-carboxylic acid, citrate, hippuric acid, glutamate and palmitic acid [131,132]. Taken together, these data suggest an increased glycine conjugation of benzoic acid to hippuric acid and an enhanced metabolism of tryptophan to indole-3-carboxylic acid in CCl_4_-induced fibrosis. Another investigation administered DMN to rats and categorized the resultant fibrosis based upon the aspartate aminotransferase (AST) level. Uric acid, orotic acid, phenacetylglycine and glutaric acid were biomarkers for the moderately high AST group, and aminomalonic acid was a biomarker for the significantly higher AST group. Arabitol distinguished fibrosis from control groups, irrespective of the AST level [133]. Another investigation used metabolic networks to analyze the urinary data after the administration of CCl_4_ to rats. They identified six key metabolites in the liver fibrosis network—glycine, glutamic acid, serine, glutamine, pyruvate and ammonia [134]. Another group applied an integrated metabolomic and proteomic investigation to the analysis of CCl_4_ administration to rats. Glucose, mannose, glycine, serine, ornithine, valeric acid, eicosenoic acid and purine were elevated in liver tissue, while acetic acid, ribonic acid, alanine, putrescine, dodecanoic acid and phosphoric acid were all depleted. Gypenoside, a saponin extract derived from *Gynostemma pentaphyllum*, was used to reverse the fibrosis [135]. The structure of gypenoside XVII, an active constituent [141] of the one hundred gypenosides known. Another study administered CCl_4_ to mice and applied a popular edible fungus *Flammulina velutipes* to reverse the fibrosis [136]. The active principle of the mushroom is not known, although recent work has identified five steroid constituents, including herbarulide and dankasterone A [142]. Six metabolites in liver were elevated in the fibrosis group, namely, citrate, stearate, glutamine, malonate, malate and proline. Ten hepatic metabolites were diminished, namely, galactose, glycerol, phosphate, glycine, acetate, glucose, gluconate, oxaloacetate, propionate and palmitate. Pathway analysis showed that the affected pathways of greatest impact and statistical significance were glyoxylate and decarboxylate metabolism, galactose metabolism, TCA cycle and alanine, aspartate and glutamate metabolism [136]. This same group used their mouse/CCl_4_ model to examine the reversal of fibrosis by Forsythiae fructus, the dried fruit of *F. suspensa* [137]. A total of 321 chemical constituents of Forsythiae fructus have been identified, all of which have been cataloged [143]. No metabolic changes were measured in this investigation. However, Forsythiae fructus was analyzed by high-performance liquid chromatography for the presence of forsythiaside A and forsythin, which are measures of the quality of Forsythiae fructus according to the Chinese Pharmacopoeia [144]. This same group again used their mouse/CCl_4_ model to examine the reversal of fibrosis by amarogentin, a secoiridoid glycoside from gentian root. Nine murine metabolites were diminished in the serum of the fibrotic group, isoleucine, threonine, β-alanine, adipic acid, 3-hydroxy-3-methylglutaric acid, phenylalanine, indolelactic acid, 5-hydroxyindoleacetic acid and arachidonic acid [138]. These findings indicate increased amino acid metabolism in fibrosis, especially tryptophan, phenylalanine, threonine and isoleucine. Increased tryptophan metabolism was observed by other workers [131,132]. In another investigation, an aqueous ethanolic extract of the flowers of Japanese honeysuckle (*Lonicera japonica*) was used to reverse fibrosis in rats treated with DMN. They reported that three urinary metabolites were increased in the fibrotic group, namely, 8-phenyl-8-azbicyclo[4.3.0]non-3-ene-7,9-dione, 2-(6-heptynyl)-1,3-dioxolane and bis(*O*-methyloxime)-4-ketoglucose) [139]. This last compound appears to be a methoximated derivative of 4-ketoglucose (the oxidation product of glucose in Fehling’s test [145]), usually formed prior to GC–MS analysis by treating the samples with methoxyamine hydrochloride. Apparently, these authors did not employ a methoximation of their samples. These findings, therefore, appear to be questionable. 

GC–MS metabolomics was also used to investigate patients with liver disease, specifically CHB [146], which, as discussed above, can lead to liver fibrosis [112]. The purpose of this study was to elucidate the structure and function of the gut microbiota in early-stage CHB and to understand its influence on disease progression [146]. A total of 85 CHB patients with low Child–Pugh scores [147] and 22 healthy controls collected their stools, which were analyzed for microbial composition. In addition, 40 serum samples were analyzed by GC–MS metabolomics [146]. Compared with the controls, significant alteration in the gut microbiota was observed in the CHB patients: 5 operational taxonomic units (O-TUs) belonging to *Actinomyces*, *Clostridium sensu stricto*, unclassified *Lachnospiraceae* and *Megamonas* were increased, and 27 belonging to *Alistipes*, *Asaccharobacter*, *Bacteroides*, *Butyricimonas*, *Clostridium* IV, *Escherichia/Shigella*, *Parabacteroides*, *Ruminococcus*, unclassified Bacteria, unclassified *Clostridiales*, unclassified *Coriobacteriaceae*, unclassified *Enterobacteriaceae*, unclassified *Lachnospiraceae* and unclassified *Ruminococcaceae* were decreased. Serum GC–MS metabolomics uncovered microbiome-specific metabolic changes in CHB. In particular, four OTUs, OTU38 (*Streptococcus*), OTU124 (*Veillonella*), OTU224 (*Streptococcus*) and OTU55 (*Haemophilus*), had high correlations with the hosts’ hepatic function indices and certain elevated serum metabolites, including phenylalanine and tyrosine [146], aromatic amino acids that have long been known to play pathogenic roles in liver disease [148,149]. Interestingly, no changes in the BCAA were found in this study despite their reported attenuation in liver disease relative to AAA (Fischer’s ratio BCAA/AAA) [149,150] and the observation that BCAA supplements are of therapeutic benefit in cirrhosis and HCC patients [151]. BCAAs are known to regulate the gut microbiota, with BCAA dietary supplementation increasing *Bifidobacterium* abundance [152]. Importantly, BCAAs are produced by several bacterial members of the gut microbiota [153]. A metabolomic and metagenomic investigation was conducted on 99 MASLD patients administered resistant starch (RS; an undigested prebiotic that feeds bacteria in the colon), and 97 MASLD controls administered regular starch in a 4-month randomized placebo-controlled clinical trial. The RS supplement resulted in decline in BCAA serum levels that correlated with a reduced fecal abundance of *Bacteroides stercoris*, *Bacteroides salyersiae*, *Parabacteroides merdae* and a *Megasphera* unclassified species. The greatest correlation was with *Bacteroides stercoris*, which is presumably the principal producer of BCAA in the gut microbiome. Moreover, the RS intervention resulted in a 9% absolute decline in intrahepatic triglyceride levels. That BCAA contributed to MASLD was further demonstrated using fecal transplantation and *B. stercoris* gavage in mice with metabolomic and metagenomic readouts [154]. These findings that arose from contemporary metabolomic and metagenomic methodologies added more detail to the association between gut microbiota, BCAA and MASLD. Plasma metabolomics found 106 endogenous metabolites altered in MASLD [155], with certain metabolic pathways related to liver fibrosis through the FIB-4 index [156].

A comprehensive two-dimensional gas chromatography–time-of-flight mass spectrometry (GC × GC–TOFMS) targeted metabolomic investigation was conducted to separate and quantitate the amino acid D- and L-enantiomers. The analysis of the serum of 25 cirrhotic patients and 16 healthy controls revealed statistically significantly elevated concentrations of D-alanine and D-proline [157]. These are two of four D-amino acids explicitly synthesized by the gut microbiota in specific pathogen-free (SPF) mice [158]. For the L-amino acids in cirrhotic serum, L-alanine, L-valine, L-isoleucine, L-leucine, L-serine and L-asparagine were all statistically significantly reduced in concentration. Typically, the L-amino acids were found at ~150-fold greater serum concentration than the D-amino acids [157]. The finding that the BCAA L-valine, L-isoleucine and L-leucine were reduced in the serum of cirrhotic patients was consistent with other observations cited above. D-amino acid oxidase (DAO) is found in intestinal epithelial cells including goblet cells, which secrete the enzyme into the lumen. DAO converts these D-amino acids into the antimicrobial product H_2_O_2_, which protects the mucosal surface of the small intestine from *Vibrio cholerae* and *V. parahaemolyticus*, diarrheal pathogens that colonize the small intestine and cause cholera and enteritis, respectively [158]. The GC–MS metabolomic study that identified statistically significantly elevated concentrations of D-alanine and D-proline in the serum of cirrhotic patients [157] points to an as yet to be investigated pathology of liver cirrhosis: attenuated small intestinal DAO activity. Furthermore, another GC–MS metabolomic study with pathway enrichment analysis followed by qPCR examination of specific gene expression reported that DAO was a candidate gene in the progression of cirrhosis to HCC. In fact, stages 1 to 3 of HCC tumor material had a lower expression of DAO than surrounding nontumorous liver tissue [159]. Intestinal DAO was not investigated. These reports underscore the value of metabolomic investigations in studying the pathobiology of liver diseases.

In the investigation of liver disease, GC–MS has sometimes been combined with other analytical platforms such as ultraperformance liquid chromatography–electrospray ionization–quadrupole time-of-flight mass spectrometry (UPLC–ESI–QTOFMS) and ultraperformance liquid chromatography–electrospray ionization–triple quadrupole mass spectrometry (UPLC–ESI–TQMS). Ours was the first investigation to combine these three platforms to study liver disease, including HCC and LC. Four groups were investigated, healthy volunteers, LC and HCC patients and a control group with acute myeloblastic leukemia (AML). Using GC–MS, we found that lignoceric acid (24:0) and nervonic acid (24:1) were virtually absent from HCC plasma compared to the other three groups. Using UPLC–ESI–TQMS, we also determined that HCC plasma had reduced concentrations of 12 lysophosphocholines (LPCs). Biliverdin, bilirubin, glycodeoxycholate, deoxycholate 3-sulfate and the fetal bile acids 7α-hydroxy-3-oxochol-4-en-24-oic acid and 3-oxochol-4,6-dien-24-oic acid were all elevated in the plasma of HCC patients [50]. We subsequently reported using UPLC–ESI–TQMS that these two fetal bile acids increased in serum of cirrhotic patients in relation to their Child–Pugh or MELD scores [160]. These fetal bile acids are known to arise in neonates with severe cholestatic liver disease due to a recessive inborn error of Δ^4^-3-oxo-steroid 5β-reductase due to mutated *AKR1D1* [161,162]. The role of fetal bile acids in adult liver disease remains to be elucidated. In an investigation of chronic liver disease due to HCV, serum lipidomics was conducted using UPLC–ESI–QTOFMS, and serum amino acid profiles were determined by GC–MS in patients with three grades of fibrosis, “low”, “mild” and “severe.” Reduction in ceramides 18:1/22:0, 18:1/24:0, diacylglycerol 42:6 and increased phosphocholine 40:6 were associated with greater fibrosis. Among the amino acids measured, only BCAA and AAAs were associated with the severity of fibrosis [163]. 

**Figure 1:**
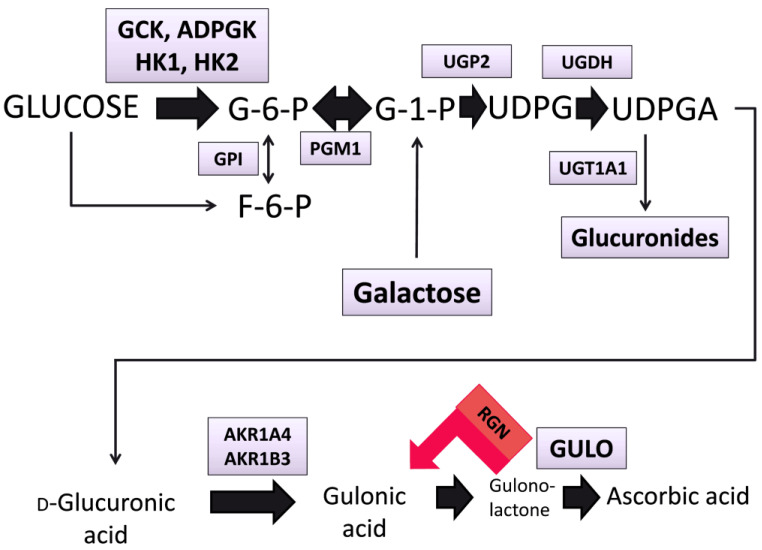
The de novo synthesis of ascorbic acid showing potential enzymes involved whose mRNA expression was determined using quantitative RT-PCR. Intermediates are G-6-P, glucose 6-phosphate; F-6-P, fructose 6-phosphate; G-1-P, glucose 1-phosphate; UDPG, uridine diphosphate glucose; UDPGA, uridine diphosphate glucuronic acid. The enzymes are GCK, glucokinase (HK4; EC 2.7.1.1); ADPGK, ADP-dependent glucokinase (EC 2.7.1.147); HK1, hexokinase 1 (EC 2.7.1.1); HK2, hexokinase 2 (EC 2.7.1.1); GPI, glucose 6-phosphate isomerase (EC 5.3.1.9); PGM1, phosphoglucomutase 1 (EC 5.4.2.2); UGP2, UDP-glucose pyrophosphorylase 2 (EC 2.7.7.9); UGDH, UDP glucose 6-dehydrogenase (EC 1.1.1.22); UGT1A1, UDP glucuronosyltransferase family 1 member A1 (EC 2.4.1.17); AKR1A4, aldo-keto reductase family 1, member A1 (aldehyde reductase; EC 1.1.1.2); AKR1B3, aldo-keto reductase family 1, member B3 (aldose reductase; EC 1.1.1.21); RGN, regucalcin (gluconolactonase; EC 3.1.1.17); GULO, gulonolactone oxidase (EC 1.1.3.8). Adapted from [164] with permission.

The most comprehensive GC–MS metabolomic study of liver fibrosis and its mechanism was conducted in mice using three independent experimental models [164]. This investigation was predicated upon the assumptions that:

(i) The stabilization of the collagen triple helix under physiological conditions is dependent upon the post-translational modification by proline 4-hydroxylation at -X-Pro-Gly sequences that require Fe^2+^, O_2_, ascorbate and 2-oxoglutarate, the last of which undergoes oxidative decarboxylation to succinate [165].
(ii) In most mammals, excluding humans, certain primates and the guinea pig, ascorbate must be synthesized de novo, and the synthetic pathway involves the conversion of glucuronic acid via gulonic acid and gulonolactone, as depicted in Figure 1. Glucuronic acid is itself synthesized from the hexose precursors glucose, fructose and galactose as shown.

We administered the hepatotoxins CCl_4_ and TAA or a 60% high-fat, choline-deficient, amino-acid-defined diet (HFCDAA) to male C57BL/6J mice. Livers collected at different times were analyzed by GC–MS metabolomics. Typical rodent metabolomic studies on fibrosis have been conducted using serum or urine, but, in our investigation, hepatic metabolite levels were determined directly using GC–MS [164].

RNA was extracted from liver and assayed by qRT-PCR for the mRNA expression of 11 genes potentially involved in the synthesis of ascorbic acid from hexoses, *Gck*, *Adpgk*, *Hk1*, *Hk2*, *Ugp2*, *Ugdh*, *Ugt1a1*, *Akr1a4*, *Akr1b3*, *Rgn* and *Gulo* (Figure 1). We reported that three mechanistically distinct liver disease models with progressive scarring in mice shared a distinct metabolic reprogramming signature in the liver that was consistent with the diversion of glucose, fructose and galactose metabolism towards the synthesis of ascorbic acid across experimental liver fibrosis models, regardless of etiology. Moreover, the increased flux in this pathway was mediated predominantly by the increased transcription of the aldose reductase *Akr1b3*. Therefore, GC–MS metabolomics was able to demonstrate that liver fibrosis in the mouse had congruent metabolic footprints, irrespective of the hepatotoxic protocol employed, and utilized the metabolic hijacking of hexose metabolism to ascorbate synthesis. Specifically, for mice treated with CCl_4_, elevated hepatic metabolites were glycerol-3-phosphate, malate, fumarate and succinate (the product of proline 4-hydroxylation). The depleted hepatic metabolites were ribose-5-phosphate, gluconic acid, glucose-6-phosphate, glucose, fructose, galactose, galactose-1-phosphate and threonic acid. For mice treated with TAA, elevated hepatic metabolites were ascorbate (the cofactor for proline 4-hydroxylation), malate, fumarate and succinate. The depleted hepatic metabolites were ribose-5-phosphate, xylose, maltose, fructose, glucose, galactose, glucuronic acid, palmitic acid, oleic acid and linoleic acid. For mice treated with a high-fat, choline-deficient amino-acid-defined diet (HF-CDAA), the elevated hepatic metabolite was ascorbate, and the depleted hepatic metabolites were glucose-6-phosphate, glycerol-3-phosphate, fructose-6-phosphate, glucose, fructose, galactose, galactose-1-phosphate, glucuronic acid, citrate, palmitic acid and oleic acid. These findings were consistent with all three fibrogenic procedures, causing an increased flux from the hexoses glucose, fructose and galactose via the glucuronic acid pathway to ascorbate and away from the pentose phosphate pathway. This hijacking of hexose metabolism to ascorbate via glucuronic acid generates an essential cofactor for the post-translational modification of collagen fibers. Before our study [164], earlier investigations failed to observe these metabolic hijacking pathways that occur in fibrosis, due to the analysis of only serum and/or urine. Moreover, and importantly, all these pathways were observed to reverse when mice were switched from the HF-CDAA to a normal diet [164]. 

### 2.6. Summary of GC–MS Metabolomic Studies of Liver Fibrosis

A significant number of investigators have employed GC–MS metabolomic methodologies to examine metabolic alterations in liver, serum or urine of rats and mice treated with fibrogens, such as CCl_4_, TAA, DMN and dietary modification. In the studies outlined above [131,132,133,134,135,136,137,138,139,140], the common upregulated metabolites in fibrosis were citrate, serine, glutamate and glutamine, and the common downregulated fibrosis metabolites were glycine and inorganic phosphate. The BCAA and AAA did not feature regularly in these studies. Several key features of the metabolic hijacking of hexose metabolism to ascorbate synthesis [164] were apparent in these other investigations [131,132,133,134,135,136,137,138,139,140], including depleted fatty acids (dodecanoic acid, palmitic acid and arachidonic acid), reflecting the feeding of the TCA cycle by β-oxidation [164] and galactose, glucose and gluconic acid. Despite the plethora of disparate observations in the aforementioned GC–MS investigations of fibrosis, a pattern has emerged, that of depleted sugars and fatty acids with an increase in the amino acids serine, glutamate and glutamine. These metabolites have not been evaluated as biomarkers for liver fibrosis but may be thought of as a gateway to an enhanced understanding of the mechanism of fibrogenesis. 

### 2.7. Liquid Chromatography–Mass Spectrometry-Based Metabolomics

The liquid chromatography–mass spectrometry (LC–MS) instrumental methodologies using UPLC–ESI–QTOFMS and UPLC–ESI–TQMS were discussed briefly above. In the past 10–15 years, there has been a gradual change from NMR-based metabolomics to LC–MS-based metabolomic studies. With regard to liver fibrosis, Chinese researchers have employed LC–MS metabolomics, like GC–MS metabolomics (see above), to define the metabolic changes in rats or mice administered CCl_4_, TAA or DMN and the metabolic effects of TCM in preventing or ameliorating the resultant experimental liver fibrosis [166,167,168,169,170,171,172]. In similar experiments where rats were administered CCl_4_, LC–MS metabolomics was employed to track the metabolic changes occurring during fibrosis or its progression [173,174]. These nine investigations in mice and rats administered either TAA, CCl_4_ or DMN yielded an abundance of metabolic data associated with the occurrence and/or progression of liver fibrosis (Table 3). 

**Table 3:** Biochemical changes provoked in rodents by various fibrogens and discovered by LC–MS.

Species	Fibrogen	Treatment	Tissue	Analytical Platform	Biochemical Pathways Affected by Fibrogen	Ref.
mouse mouse rat rat mouse rat mouse rat rat	TAA CCl_4_ CCl_4_ CCl_4_ CCl_4_ DMN CCl_4_ CCl_4_ CCl_4_	picroside I phylligenin TACS Ganlong capsules YQJPF FHC curcumol - - -	serum urine liver feces feces liver serum urine feces serum serum urine serum serum	UPLC–QTOFMS GC–MS UPLC–TQMS UPLC–QTOFMS LC–MS LC–MS/MS LC–MS LC–MS UPLC–QTOFMS UPLC–QTOFMS	(PC, LPC) ↑ valine ↑ (glycerophospholipid metabolism, GSH, GSSG) ↑ SCFA ↓ BA (alloLCA, LCA, isoLCA, 7-ketoLCA, norDCA, CDCA, UDCA, HDCA, norCA, 6,7-diketoLCA, α-MCA, UCA, β-MCA; TCDCA) ↑ (5-phosphonooxy-L-lysine, 5-hydroxy-L-tryptophan, phosphoserine, 7-methylinosine, 3-methyl-2-(3-pyridyl)-1-indoleoctanoic acid *, β-alanyl-L-lysine, GCA, KCA, 7-sulfocholic acid, homomethionine, 3-methylindole, benzphetamine *) ↑ (glutathione, deoxyinosine, γ-glutamylglutamic acid, *N*^2^-acetyl-ornithine, CA, KDCA, CDCA, NCA, DCA, lysyltyrosine, *N*-acetyl-L-methionine) ↓ (5α-pregnane-3,20-dione, 4-(4-methyl-3-pentenyl)-3-cyclohexene-1-carboxaldehyde), PG(18:2(9*Z*,12*Z*)/18:2(9*Z*,12*Z*)), 6-hydroxy-1*H*-indole-3-acetamide, vanilloside, PC(20:4(8*Z*,11*Z*,14*Z*,17*Z*)/*P*-18:0), PE(16:0/18:3(9*Z*,12*Z*,15*Z*)), 5-methylcytidine, L-carnitine, 2-hydroxy-6-pentadecylbenzoic acid **, *m*-coumaric acid, imidazoleacetic acid, uracil, prostaglandin E2, deoxycytidine, (R)-3-hydroxybutyric acid, pyroglutamic acid, γ-aminobutyric acid, *myo*-inositol, *O*-phosphoethanolamine) ↑ (DG(18:1(9*Z*)/18:4(6*Z*,9*Z*,12*Z*,15*Z*)), xanthosine, xanthine, *N*-acetylhistidine, β-D-glucosamine, *N*^6^-acetyl-L-lysine, γ-glutamylleucine, ascorbic acid, eicosapentaenoic acid, acetylglycine, citraconic acid, deoxyribose 5-phosphate, dihydrolipoate, prostaglandin B1, D-ribose, 2-furoic acid, isobutyrylglycine, L-iditol, fructose 1-phosphate, 2-ketobutyric acid, citramalic acid, D-mannose, *S*-adenosylhomocysteine) ↓ (thymine, PC(P-18:1(11*Z*)/22:5(4*Z*,7*Z*,10*Z*,13*Z*,16*Z*)), PE(*P*-18:1(11*Z*)/22:5(4*Z*,7*Z*,10*Z*,13*Z*,16*Z*)), PC(20:2(11*Z*,14*Z*)/20:4(5*Z*,8*Z*,11*Z*,14*Z*)), PE(*P*-18:1(9*Z*)/ 20:4(5*Z*,8*Z*,11*Z*,14*Z*)), PE(*O*-16:1(1Z)/22:6(4*Z*,7*Z*,10*Z*,13*Z*,16*Z*,19*Z*)), PC(20:4(8*Z*,11*Z*,14*Z*,17*Z*)/P-18:0), vaccenylcarnitine, SM(d16:1/24:1(15*Z*)), (*E*)-5-tetradecanoylcarnitine, dodecanoylcarnitine, trimethylamine *N*-oxide, lysylvaline, hexylresorcinol *, maslinic acid *, thymidine, *p*-cresol) ↑ (dieporeticenin, 1*H*-indole-3-carboxaldehyde, 2-furoic acid, malonic acid) ↓ ginkgolide B * ↑ (dimethylhistamine, adrenochrome, *N*^2^-methylnorsalsolinol, vanillylamine, meconine, homovanillin, 3-methylpyrrolo[1,2-a]pyrazine *, β-D-glucosamine, isopentyl β-D-glucoside *, 2-methylbutyroylcarnitine, 4-trimethylammoniobutanoic acid, alanylproline, choline, naphthalene epoxide *, 2-acetylthiazole **, hydroxyprolylleucine, L-leucine, betaine aldehyde, lauroyl diethanolamide *) ↓ (4,4′-methylenebis(2,6-di-*tert*-butylphenol) *, mono(2-ethylhexyl)phthalate *, 2,3-dinor prostaglandin E1, mozenavir *, *p*-cresylsulfate) ↑ Paper in Chinese (soyasaponin I *, guggulsterone *, 3-ureidopropionic acid, 7-methylguanine, carnitine, *N*-acetylglutamic acid, *N*′^2^-benzylidene-5-hex-1-ynylfuran-2-carbohydrazide *, 3β,7β-dihydroxy-5-androsten-17-one, 4-oxoretinol, (*Z*)-9,10,11-trihydroxyoctadec-12-enoic acid *, 3-acetyl-11-keto-β-boswellic acid *, avocadyne 1-acetate, propionylcarnitine, 12,13-epoxy-9-octadecenoic acid, *N*-methylhydantoin ^‡^, 4-hexyloxyaniline *, methionine sulfoxide, aspartylglutamate, α-lapachone *, pantethine, *O*-acetylserine, 3-hydroxy-2-(3-nitro-4-piperidenylbenzyl)propanenitrile *, PC(4:0/18:5), PC(18:3/3:0), PC(14:1/24:1), PC(14:1/3:0), N^5^-(1,3,6-trimethyl-1*H*-pyrazol-4-yl)-1*H*-1,2,4-triazole-3,5-diamine *, histamine, glycyltyrosine, MAG(18:2), 2-deoxyuridine, 4-methyl-5-oxo-2-pentyl-2,5-dihydrofuran-3-carboxylic acid (striatisporolide A) *, cytidine, valproic acid *, 6-ketoprostaglandin F1α, indole-5,6-quinone) ↑ (methionine, leukotriene C4, norvaline, *N*-isobutyrylglycine, theophylline *, D-ala-D-ala, dihydroroseoside *, arginine, PE(16:0/22:6), cinnamoylglycine, pyroglutamic acid, isoniazid *, 5-[(10*Z*)-14-(3,5-dihydroxyphenyl)tetradec-10-en-1-yl)benzene-1,3-diol*, trimethyllysine, LPE(22:4)) ↓ (tryptophan, *cis*-aconitic acid, methylmalonic acid) ↑ (kynurenic acid, 5-hydroxyindoleacetylglycine, 3-methyldioxyindole, 4-(2-amino-3-hydroxyphenyl)-2,4-dioxobutanoic acid, isocitric acid, leucine) ↓ (valine, leucine, tryptophan, cholesterol, GCA) ↑ (sphinganine, lactosylceramide, sphingomyelin, lysoPC(17:0), PC (18:1(11*Z*)/20:5(5*Z*,8*Z*,11*Z*,14*Z*,17*Z*)) ↓ (β-MCA, cervonoyl ethanolamide, hydroxyethyl glycine, threonine, indoleacetic acid) ↑	[166] [167] [168] [169] [170] [171] [172] [173] [174]

**Footnotes:** Direction of arrows (↑↓) indicates metabolic changes provoked by the fibrogen; * = not endogenous but from exposome [175]; ** = probably in rodent diet; ^‡^ = likely gut microbiota metabolite. Abbreviations: UPLC–QTOFMS, ultraperformance liquid chromatography–quadrupole time-of-flight mass spectrometry; SCFA, short-chain fatty acids; BA, bile acids; LCA, lithocholic acid; isoLCA, isolithocholic acid; 7-ketoLCA, 7-ketolithocholic acid; DCA, deoxycholic acid; CDCA, chenodeoxycholic acid; UDCA, ursodeoxycholic acid; HDCA, hyodeoxycholic acid; norCA, norcholic acid; 6,7-diketoLCA, 6,7-diketo-lithocholic acid; MCA, muricholic acid; UCA, ursocholic acid; β-MCA, β-muricholic acid; TCDCA, taurochenodeoxycholic acid; UPLC–TQMS, ultraperformance liquid chromatography–triple quadrupole mass spectrometry; TACS, total alkaloids of *Corydalis saxicola* Bunting; GCA, glycocholic acid; KCA, ketocholic acid; CA, cholic acid; KDCA, ketodeoxycholic acid; NCA, nutriacholic acid; YQJPF, Yi–Qi–Jian–Pi formula; DMN, dimethylnitrosamine; FHC, Fuzheng Huayu capsule.

**Figure 2:**
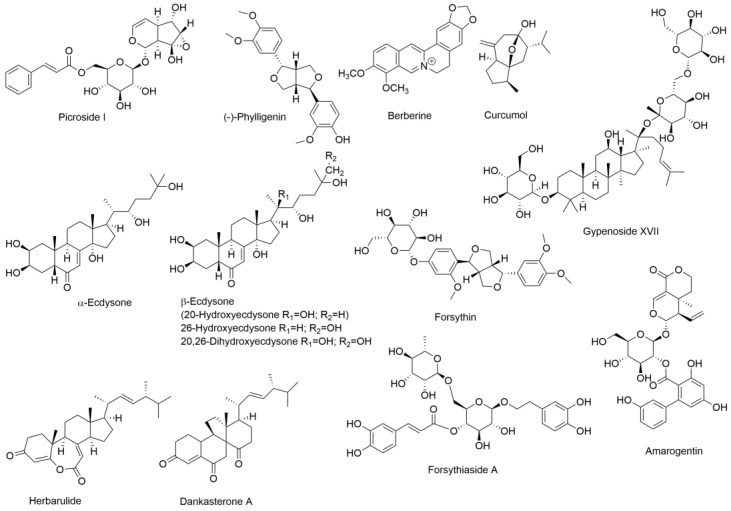
The treatments or the chemical constituents contained therein for experimental fibrosis in rodents. Picroside I [166] and (–)-phylligenin [167] were administered as such. Berberine is one of the isoquinoline alkaloids present in *Corydalis saxicola* Bunting [168]. The ecdysone insect molting hormones are likely significant constituents of the ethanol extract of *Periplanata americana* (American cockroach) that comprises Ganlong capsules [169]. Gypenoside XVII is a saponin extract derived from *Gynostemma pentaphyllum* [135,141]. Amarogentin is a secoiridoid glycoside from gentian root [138]. Herbarulide and dankasterone A are from the popular edible fungus *Flammulina velutipes* [136]. Forsythin and forsythiaside A are from Forsythiae fructus [137].

The first investigation in mice employed picroside I ([(2*R*,3*S*,4*S*,5*R*,6*S*)-3,4,5-trihydroxy-6-[[(1*S*,2*S*,4*S*,5*S*,6*R*,10*S*)-5-hydroxy-2-(hydroxymethyl)-3,9-dioxatricyclo[4.4.0.02,4]dec-7-en-10-yl]oxy]oxan-2-yl]methyl (*E*)-3-phenylprop-2-enoate) (Figure 2), a hepatoprotectant isolated from *Gentiana kurroo*, *Picrorhiza kurroa* and *P. scrophulariiflora*, administered to mice after treatment with TAA and used to generate fibrosis [166]. Table 3 shows the power of LC–MS platforms with the metabolomic analysis of serum, urine and liver. Multiple metabolic routes were upregulated by picroside I administration, indicating that these pathways were downregulated in TAA-induced fibrosis [166]. These included various lipid, amino acid and energy metabolism pathways. 

The second investigation also in mice used phylligenin (4-[(3*R*,3*aS*,6*S*,6*aS*)-6-(3,4-dimethoxyphenyl)-1,3,3*a*,4,6,6*a*-hexahydrofuro [3,4-c]furan-3-yl]-2-methoxyphenol) (Figure 2), a lignan from the fruits of *Forsythia koreana* with anti-inflammatory properties [176]. After CCl_4_ was administered to mice to generate fibrosis, short-chain fatty acid (SCFA) levels were depressed, and bile acids were elevated in the gut [167]. These findings show that liver fibrosis in the mouse is associated with an increase in bile acid synthesis in the liver and a decrease in SCFA production in the gut. 

The third investigation conducted in rats used the total alkaloids of *Corydalis saxicola* Bunting (TACS) in an attempt to reverse the fibrosis generated by the administration of CCl_4_. TACS comprises a mixture of related isoquinoline alkaloids, including cheilanthifoline, berberrubine, epiberberine, tetrahydropalmatine, jatrorrhizine, coptisine, dehydrocavidine, palmatine, berberine and chelerythrine [168]. The chemical structure of berberine, for example, is shown in Figure 2. The metabolomic analysis of rat fecal extracts using UPLC–QTOFMS revealed a panoply of both elevated and diminished metabolites caused by the generation of fibrosis by CCl_4_ (Table 3). The affected metabolic pathways largely involved amino acid, purine and bile acid metabolism. One major difference in these findings [168] compared to the report of CCl_4_ and phylligenin administered to mice [167], both studies using LC–MS to analyze feces, is the reported bile acid profile (Table 3). This may reflect a species difference between the mouse and the rat. While the mouse possesses a gall bladder, the rat does not [177,178]. The rat produces a more concentrated bile than most other species and, therefore, has no need for a gall bladder to concentrate the primary bile. Moreover, the synthesis and conjugation of bile acids produce considerably different bile acid profiles between the mouse and rat [179].

The fourth investigation was carried out in rats administered CCl_4_. The TCM treatment used to reverse fibrosis was Ganlong capsules, a preparation made from an ethanol extract of *Periplanata americana* (the American cockroach) [169]. The perusal of reference [180] indicates that *Periplanata americana* generates the molting hormone α-ecdysone, which is highly soluble in ethanol (20 mg/mL) [181]. It may be speculated that α-ecdysone (Figure 2) is responsible for the putative hepatoprotective effects of Ganlong capsules. However, α-ecdysone undergoes 20-hydroxylation to β-ecdysone (Figure 2), followed by 26-hydroxylation [182]. There is evidence that β-ecdysone may be the active insect molting hormone [183,184] with 100× potency of the prohormone α-ecdysone in a *Drosophila melanogaster* assay in vitro [185]. In the rats treated with CCl_4_ and then Ganlong capsules, an analysis of liver, serum and urine was conducted by LC–MS, although the instrument used was not described [169]. Table 3 shows the plethora of metabolites found to be elevated or depressed relative to controls in liver, serum and urine for animals treated with CCl_4_. It is difficult to compare these findings to another study that administered CCl_4_ to rats [168], as these latter investigators only analyzed feces, not liver, serum or urine. 

The fifth investigation administered CCl_4_ to mice and employed Yi–Qi–Jian–Pi formula (YQJPF) to reverse the experimental liver fibrosis. YQJPF comprises Huangqi (*Astragalus propinquus*), Taizishen (*Pseudostellaria Radix*), Baizhu (*Atractylodis Macrocephalae Rhizoma*), Chenpi (*Pericarpium Citri Reticulatae*), Danggui (*Radix Angelica Sinensis*), Fulin (*Sclerotium Poriae Cocos*), Huangqin (*Scutellaria baicalensis Georgi*) and Gancao (*Radix Glycyrrhizae*). The pharmacologically active chemical constituent of YQJPF is not known. Metabolites in feces were determined by LC–MS/MS by a contract lab, and, therefore, further details were not given. The five most “differential metabolites” between CCl_4_-treated and control mice feces surprisingly comprised two endogenous metabolites (2,3-dinor prostaglandin E1 and *p*-cresylsulfate) but also three exogenous molecules (4,4′-methylenebis(2,6-di-*tert*-butylphenol), mono(2-ethylhexyl)phthalate and mozenavir (an experimental antiviral agent for the treatment of AIDS)). It is not easy to imagine how these three foreign chemicals were found in mouse feces. Perhaps they were accidentally introduced into the samples by the analytical contractor. This report does not inform the mechanism of CCl_4_ fibrogenesis in the mouse.

The sixth study involved the administration of dimethylnitrosamine (DMN) to generate fibrosis in the rat. To reverse the fibrosis, the TCM Fuzheng Huayu capsule (FHC) was given [171] (Table 3). FHC is a Chinese herbal formula against liver fibrosis. It comprises six components, namely, *Savia miltiorrhiza*, *Cordyceps sinensis*, *Gynostemma pentaphyllum*, pine pollen, *Prunus persica* and *Schisandra chinensis* [186]. This paper is in Chinese, so no further details were discernable.

The seventh investigation administered CCl_4_ to mice and investigated the efficacy of curcumol (Figure 2) in reversing fibrosis [172]. Using LC–MS with a high-resolution Orbitrap mass spectrometer, these authors describe a large number of metabolites both up- and downregulated. Four phosphatidylcholines were elevated, and one phosphatidylethanolamine and one lysophosphatidylethanolamine were depressed in serum (Table 3). Altered phospholipid metabolism was also observed in the liver and serum of rats with liver fibrosis that had been administered CCl_4_ [169] and in the serum and liver of mice with fibrosis administered TAA [166]. The rate-limiting step for phosphatidylcholine synthesis is catalyzed by CTP:phosphocholine cytidylyltransferase α (CCTα), which is found in the nucleus in most cell types [187]. How CCTα might be involved in fibrogenesis is uncertain. Surprisingly, a considerable number of the features reported in this study were exogenous chemicals, such as soyasaponin I, guggulsterone, *N*’^2^-benzylidene-5-hex-1-ynylfuran-2-carbohydrazide, 3-acetyl-11-keto-β-boswellic acid, 4-hexyloxyaniline, α-lapachone and 3-hydroxy-2-(3-nitro-4-piperidenylbenzyl)propanenitrile, to name but a few. 4-Hexyloxyaniline, for example, has a long aliphatic chain resembling certain quorum sensing and autoinducer molecules produced by bacterial species in the gut microbiota [188]. However, this is where the similarity ends. 3-Acetyl-11-keto-β-boswellic acid is derived from *Boswellia serrata*, the tree that produces Indian frankincense [189,190]. Similarly, guggulsterone is derived from the gum resin of *Commiphora wightii* and has been used for a thousand years in Ayurvedic medicine [191]. It is hard to conceive how laboratory mice housed under sterile conditions with filtered air could be exposed to such a wide range of xenobiotics. The authors stated that they had found 54 differentially expressed metabolites between the control and CCl_4_-treated animals. Unfortunately, approximately half of these features may be spurious. 

The eighth study was a metabolomic investigation on the urine and serum of the progression of liver fibrosis after the administration of CCl_4_ to rats [173]. Therefore, only diagnostic biomarkers were described using UPLC–QTOFMS (Table 3). In urine, tryptophan, *cis*-aconitic acid and methylmalonic acid were increased. *cis*-Aconic acid is a TCA cycle intermediate involved in the isomerization of citrate to isocitrate, and methylmalonic acid is primarily a breakdown product of the essential amino acids methionine, valine, isoleucine and threonine, as well as of the cholesterol sidechain and branched-chain fatty acids [192]. Decreases in urine were reported in several tryptophan metabolites, kynurenic acid, 5-hydroxyindoleacetylglycine, 3-methyldioxyindole and 4-(2-amino-3-hydroxyphenyl)-2,4-dioxobutanoic acid, indicating an impaired catabolism of tryptophan during fibrosis caused by CCl_4_ in the rat. Isocitrate and leucine were also depleted in urine, consistent with the elevations in *cis*-aconic acid and methylmalonic acid, respectively. Accordingly, the metabolism of isocitrate and leucine was also impaired during this experimental fibrosis. In serum, valine, leucine, tryptophan, cholesterol and GCA were all elevated, consistent with the urinary findings. Depleted in serum were the lipids sphinganine, lactosylceramide, sphingomyelin, lysoPC(17:0) and PC (18:1(11*Z*)/20:5(5*Z*,8*Z*,11*Z*,14*Z*,17*Z*). Sphingolipid metabolism is known to be downregulated in liver fibrosis [193], as is glycerophospholipid metabolism [173]. This LC–MS-based metabolomic investigation furnished important insights into the mechanism of liver fibrosis in CCl_4_-treated rats.

The nineth and final study again employed CCl_4_ to generate liver fibrosis in the rat. As with the previous study, only diagnostic biomarkers were described using UPLC–QTOFMS [174]. Five putative serum biomarkers were reported (Table 3): β-MCA, cervonoyl ethanolamide, hydroxyethyl glycine, threonine and indoleacetic acid, of which β-MCA and cervonoyl ethanolamide were able to predict the stage of fibrosis in CCl_4_-treated rats. In addition, ten lysophosphatidylcholines and three phosphatidylcholines were found to be affected (not listed in Table 3), resulting in pathway enrichment analysis using MetaboAnalyst (www.metaboanalyst.ca, accessed on 1 March, 2024) demonstrating that glycerophospholipid metabolism was both the most impactful and statistically significant pathway affected by CCl_4_-generated liver fibrosis in the rat [174]. 

### 2.8. Summary of LC–MS Metabolomic Studies of Liver Fibrosis

As mentioned above, a common theme of the studies in Table 3 is phospholipid metabolism, with elevated phosphatidylcholines in fibrosis, whether caused by TAA or CCl_4_ in the mouse or CCl_4_ in the rat [166,169,172]. In a recent report, plasma bile acid analysis showed higher levels of GCDCA, TCDCA, GCA and TCA in patients with liver fibrosis than in normal controls. In mice, glycochenodeoxycholic acid (GCDCA) increased collagen fibers in the liver [194]. In the studies summarized in Table 3, an array of bile acids was elevated in both rat and mouse feces after the administration of CCl_4_ [167,168] consistent with the recent findings. Fischer’s ratio, the ratio of BCAA to AAA, was the only change in serum amino acids observed in patients with fibrosis, with a decrease in serum branched-chain amino acids and an increase in aromatic amino acids [163]. Furthermore, in an investigation of dogs with chronic hepatitis, Fischer’s ratio decreased in proportion to the degree of fibrosis [195]. Remarkably, in the series of investigations outlined in Table 3, no such BCAA/AAA ratio relationship was reported in any mouse or rat study. This perhaps is a weakness of the LC–MS methodologies employed in these studies, which are not centered on small polar molecules such as amino acids but rather operate best for larger molecules such as phospholipids. In order to include small, polar, hydrophilic molecules in an LC–MS metabolomic protocol, a UPLC–ESI–QTOFMS analysis of each sample is also required using a Hydrophilic Interaction Liquid Chromatography (HILIC) column [196] in addition to the standard analysis that employs a reverse-phase (RP) column. GC–MS protocols are best suited for small polar molecules, such as amino acids, sugars and TCA cycle intermediates, as we have previously discussed [197]. Apart from phospholipid and bile acid metabolism, these LC–MS investigations added few insights into the mechanisms of liver fibrosis in rodents.

## 3. Overall Summary of NMR, GC–MS and LC–MS Investigations Into Liver Fibrosis

**Figure 3:**
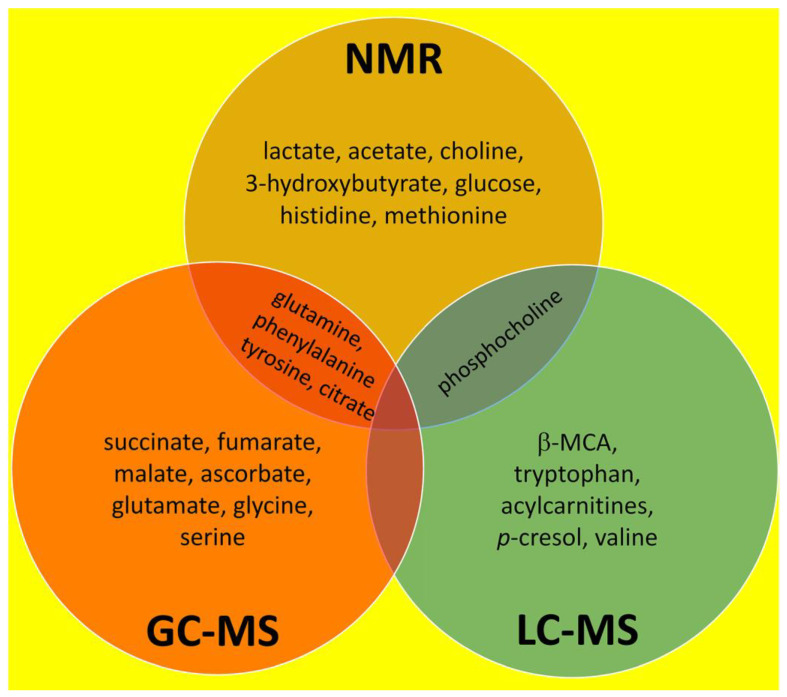
Venn diagram showing the upregulated metabolites in mouse, rat and human serum, urine, liver and feces discovered by NMR, GC–MS and LC–MS. Metabolites were included in each section if they had been reported in two or more studies. Note that no metabolites were discovered universally by all three analytical platforms or in common by GC–MS and LC–MS.

The findings of the investigations reviewed here are summarized in Table 2 and Table 3 and Section 2.4, Section 2.6 and Section 2.8. The earliest NMR studies employed a 0.0025 T magnet operating at a frequency of 100 MHz [67]. The most recent NMR studies used an 18.8 T magnet operating at 800 MHz [111]. As shown in Table 2, NMR spectroscopy-based metabolomics was a popular methodology used in clinical studies, perhaps because of its promotion by Jeremy Nicholson, the founder of “metabonomics”, [59] and former head of the Department of Surgery and Cancer, Imperial College London [198,199,200,201]. Three NMR studies in the rat that used TAA or aflatoxin B_1_ to induce fibrosis and eight clinical studies where HBV, HCV or alcohol caused liver fibrosis reported abundant upregulated metabolites in plasma, serum, liver and urine (Table 2). The details of the elevated metabolites from the NMR, GC–MS and LC–MS investigations reviewed here are shown in the Venn diagram in Figure 3. A total of 24 upregulated metabolites in three species with liver fibrosis were found: seven by NMR, seven by GC–MS, five by LC–MS, four by both NMR and GC–MS and one by both NMR and LC–MS. The advantages and disadvantages of NMR compared to chromatographic methodologies have been compared. NMR is non-destructive and inherently quantitative, with a limit of detection (LOD) of only 10^−9^ mol. By comparison, LC–MS is specific (MS/MS) with a LOD of 10^−13^ mol [202]. GC–MS analysis is slow and requires chemical derivatization of the samples. NMR can identify the 50 most abundant metabolites in plasma, GC–MS about 100, and LC–MS over one thousand metabolites. The metabolomic footprint of liver fibrosis comprised elevated glutamine, phenylalanine, tyrosine, citrate and phosphocholine (Figure 3). Glutamine is the most abundant nonessential amino acid that can be synthesized from glucose. Glutamine is converted to glutamate by glutaminase and onto 2-oxoglutarate (α-ketoglutarate) by glutamate dehydrogenase [203]. 2-Oxoglutarate is a cofactor for prolyl 4-hydroxylase where it is decarboxylated to succinate. Proline 4-hydroxylation is essential for the stabilization of the collagen triple helix [164]. Note that both elevated glutamate and succinate were discovered in fibrosis by GC–MS (Figure 3). Regarding the AAA phenylalanine and tyrosine that were discovered to be elevated in fibrosis by both NMR and GC–MS, these findings were in agreement with other work where an increased severity of fibrosis was associated with higher tyrosine, phenylalanine, methionine and citrate levels in plasma [204]. Both methionine and citrate are also included in Figure 3. The role of lipids in liver fibrosis is both a controversial and complex topic. Concerning phosphocholine, the only elevated metabolite in liver fibrosis discovered by both NMR and LC–MS, there are conflicting reports. For example, in a study of alcohol-related liver fibrosis in 315 patients and 51 matched healthy controls, 198 hepatic and 236 circulatory lipids were identified. Sphingomyelins, ceramides and phosphocholines were all downregulated in both liver and plasma, where lower abundance correlated with the severity of fibrosis [205]. That notwithstanding, it can be restated that the metabolomic footprint of liver fibrosis comprises elevated glutamine, phenylalanine, tyrosine, citrate and phosphocholine (Figure 3). 

Much more in-depth studies are clearly needed to fully elucidate and leverage the metabolic changes in liver fibrosis for diagnostic and therapeutic purposes. For instance, there is lack of studies on the profiling of metabolomes captured in extracellular vehicles (EVs), recently emerging as major players in the pathophysiology of chronic liver disease and intensely studied as diagnostic markers [206].

## 4. Brief Commentary on Medicinal Treatments for Liver Fibrosis

Many of the investigations discussed above used TCM preparations to reverse experimental liver fibrosis in rodents. The chemical structures of several active and putative constituents are given in Figure 2. The first and most remarkable point is that only one of these 12 compounds, berberine, contains a nitrogen atom. It has been estimated that 84% of small molecule drugs approved by the FDA contains at least one nitrogen atom; furthermore, 60% contain a nitrogen heterocycle [207]. These natural products used to treat liver diseases by TCM, therefore, contain an unusual group of active molecules. Two quite dissimilar molecules, phylligenin and forsythin, both from Forsythiae fructus, contain the furo[3,4-c]furan nucleus. Phylligenin was employed as a pure substance against CCl_4_-induced liver fibrosis in mice [167], and forsythin was a constituent of Forsythiae fructus water, which was used also to reverse liver fibrosis in mice [137]. However, whether or not the furo[3,4-c]furan nucleus is an active pharmacophore requires further investigation. Several of these medicinal treatments have a steroid or steroid-like structure, including gypenoside XVII, α- and β-ecdysone, herbarulide and dankasterone A (Figure 2). It is not known what receptor, transporter or enzyme is involved in the response of the fibrotic liver to any of the natural products displayed in Figure 2. If some educated guesses could be made by experimental hepatologists then medicinal chemists could test these and other natural compounds known to abrogate or reverse experimental liver fibrosis. Molecular docking studies [208,209,210,211] would be useful here, especially as it is possible to screen thousands of structures virtually in a few seconds using a standard Linux PC [210], once the target protein has been proposed. The fact that the compounds in Figure 2 range from small, such as curcumol, to large, for example, gypenoside XVII, should not impede molecular docking evaluations, as the view has been reported that binding pockets may be on average three times larger than the ligand that they bind [211]. This would permit flexibility in ligand size.

Such work is of importance given the stark absence of drugs approved to treat liver fibrosis, a disease that may affect a minimum of one-in-twenty persons worldwide [212,213,214]. It is encouraging that the TCM treatments referred to above all reduced experimental liver fibrosis in rodents, presumably by reversing or preventing oxidative stress and/or lipid peroxidation. The known or putative active chemical constituents of these TCM treatments are shown in Figure 3. They do not appear to be redox-active compounds that would simply attenuate oxidative stress or lipid peroxidation.

In contrast to experimental liver fibrosis, the first drug that may reverse liver fibrosis in patients (Rezdiffra; resmetirom) was just approved by the FDA on 14 March 2024. Rezdiffra is indicated in conjunction with diet and exercise for the treatment of adults with noncirrhotic, nonalcoholic steatohepatitis (NASH) with moderate to advanced liver fibrosis (consistent with stages F2 to F3 fibrosis). There is clinical trial evidence that Rezdiffra may reduce fibrosis by up to one stage [215], despite this not being the primary drug target. Resmetirom is a thyroid receptor-β (THR-β) partial agonist [215], and THR-β appears to be one possible starting point for molecular docking investigations with chemical constituents of TCM therapies known to reverse experimental liver fibrosis. It is hoped that research of this kind and related efforts may usher in a new era for the pharmaceutical treatment of liver fibrosis.

## Figures and Tables

**Table 1:** Summary of exposures reported to cause or prevent liver fibrosis.

Causative	Preventative
Dietary restriction [13] High-fat diet [26] Aging [14] Dietary cystine [31] Proteins rich in cystine [31] *Schistosoma mansoni* infection [15] *Schistosoma japonicum* infection [16] *Salmonella typhosa* endotoxin [25] Foreign proteins [17] Hypophysectomy [18,19] Thyroidectomy [19] Chloroform [20] Carbon tetrachloride [21] Ethionine [22] Thioacetamide [23] Galactosamine [24]	High-protein diet [26] High-carbohydrate diet [26] Casein [30] Lecithin [27] Choline [27] Methionine [30,31]

## Data Availability

No new data were created or analyzed in this study. Data sharing is not applicable to this article.
